# Cholenic acid derivative UniPR1331 impairs tumor angiogenesis via blockade of VEGF/VEGFR2 in addition to Eph/ephrin

**DOI:** 10.1038/s41417-021-00379-5

**Published:** 2021-08-23

**Authors:** Marco Rusnati, Giulia Paiardi, Chiara Tobia, Chiara Urbinati, Alessio Lodola, Pasqualina D’Ursi, Miriam Corrado, Riccardo Castelli, Rebecca C. Wade, Massimiliano Tognolini, Paola Chiodelli

**Affiliations:** 1 Experimental Oncology and Immunology, Department of Molecular and Translational Medicine, University of Brescia, Brescia, Italy; 2 Molecular and Cellular Modeling Group, Heidelberg Institute for Theoretical Studies, Heidelberg, Germany; 3 Department of Food and Drug, University of Parma, Parma, Italy; 4 Institute for Biomedical Technologies, National Research Council (ITB-CNR), Segrate, MI Italy; 5 Center for Molecular Biology (ZMBH), DKFZ-ZMBH Alliance, Heidelberg University, Heidelberg, Germany; 6 Interdisciplinary Center for Scientific Computing (IWR), Heidelberg University, Heidelberg, Germany

**Keywords:** Cell biology, Tumour angiogenesis

## Abstract

Angiogenesis, the formation of new blood vessels from preexisting ones, is crucial for tumor growth and metastatization, and is considered a promising therapeutic target. Unfortunately, drugs directed against a specific proangiogenic growth factor or receptor turned out to be of limited benefit for oncology patients, likely due to the high biochemical redundancy of the neovascularization process. In this scenario, multitarget compounds that are able to simultaneously tackle different proangiogenic pathways are eagerly awaited. UniPR1331 is a 3β-hydroxy-Δ^5^-cholenic acid derivative, which is already known to inhibit Eph–ephrin interaction. Here, we employed an analysis pipeline consisting of molecular modeling and simulation, surface plasmon resonance spectrometry, biochemical assays, and endothelial cell models to demonstrate that UniPR1331 directly interacts with the vascular endothelial growth factor receptor 2 (VEGFR2) too. The binding of UniPR1331 to VEGFR2 prevents its interaction with the natural ligand vascular endothelial growth factor and subsequent autophosphorylation, signal transduction, and in vitro proangiogenic activation of endothelial cells. In vivo, UniPR1331 inhibits tumor cell-driven angiogenesis in zebrafish. Taken together, these data shed light on the pleiotropic pharmacological effect of UniPR1331, and point to Δ^5^-cholenic acid as a promising molecular scaffold for the development of multitarget antiangiogenic compounds.

## Introduction

Angiogenesis, the formation of new blood vessels from preexisting ones, is involved in different pathologies, including cancer [1]. It results from the interaction of angiogenic growth factors (AGFs) with tyrosine kinase receptors (RTKs) exposed on the endothelial cell (EC) surface.

The vascular endothelial growth factor (VEGF) family plays a pivotal role in tumor neovascularization [2]. It comprises six subgroups: VEGFA–E and placental growth factor, with VEGFA (hereafter VEGF) mainly involved in angiogenesis. VEGFs interact differently with three distinct VEGFRs expressed on ECs, with VEGFR2 representing the primary proangiogenic receptor. The 762 amino acids VEGFR2 extracellular region is folded into seven immunoglobulin domains, with the VEGF-binding site located on domains 2 and 3 (D2–D3) [3]. Engagement by VEGF causes VEGFR2 dimerization, internalization, and the activation of a signal cascade responsible for EC proangiogenic activation and neovascularization.

VEGF/VEGFR2 proangiogenic activity depends on a complex cross talk with other receptor systems including integrins, neuropilin [2], and the erythropoietin-producing hepatocellular carcinoma receptor (Eph)/ephrin system [4], as demonstrated by the observation that the specific EphB4 kinase inhibitor NVP-BHG712 inhibits VEGF-driven angiogenesis [5] and that ephrin-B2 associates with VEGFR2 to form a complex stabilized by syntenin and required for VEGFR2 phosphorylation and downstream signaling [6].

Human Ephs constitute the largest subfamily of RTKs divided into “A” (EphA1–EphA8 and EphA10) and “B” (EphB1–EphB4 and EphB6) subclasses. They are activated upon binding to their membrane-bound cognate ephrin ligands, which induces receptor clustering, internalization, and degradation. EphA2, EphB2, and EphB4 are involved in tumor growth and neovascularization [7].

Disruption of the VEGF/VEGFR2 interaction represents an antiangiogenic strategy actively pursued in the past that has led to the development of many inhibitors, including the anti-VEGF antibody bevacizumab, currently employed in cancer therapy [8]. On the other hand, the Eph/ephrin system is an emerging target for the development of novel anticancer therapies. Indeed, monoclonal antibodies or recombinant proteins targeting the EphA2 receptor impair tumor angiogenesis and block tumors growth and metastatization in animals [9].

Unfortunately, monotherapy regimens have so far demonstrated limited clinical benefits, possibly due to the development of drug resistance and/or to the biochemical redundancy, whereby two or more AGFs play similar biochemical activities allowing each one to compensate when the other(s) are inhibited. In effect, the angiogenic process can hardly be considered the outcome of a single receptor/ligand interaction, being rather caused by the simultaneous action of multiple AGFs interacting with different proangiogenic receptors [10]. This “angiogenic interactome” is extremely intricate due to the high number of AGF families, each composed of many members that are in turn represented by different isoforms generated by alternative splicing of the transcript of a single gene [11, 12]. The “therapeutic impasse” caused by this biochemical redundancy might be overcome by using appropriate combinations of different drugs targeting different AGFs/receptors axes [13, 14]. An ambitious alternative is to develop multitarget drugs able to simultaneously tackle different proangiogenic effectors [11, 15–17].

The design of effective antagonists of protein–protein interactions (PPI) is considered a challenge in the drug discovery field [18] but provides opportunities for the development of multitarget inhibitors [16]. PPI-inhibitors (PPI-is) targeting Eph/ephrin systems [19–21] have been already described: UniPR1331 is an orally bioavailable pan-Eph PPI-i with potential for the treatment of glioblastoma [14, 22] that is also able to inhibit VEGF-induced neovascularization in vivo [14]. However, whether this VEGF inhibition was merely due to the pan-Eph PPI-i action of UniPR1331 or to a direct action on VEGFR2 is still unknown. Here, we report that the Eph/ephrin inhibitor UniPR1331 effectively binds to VEGFR2 and masks the receptor from its physiological ligand VEGF, thus exerting a promising dual antiangiogenic activity.

## Material and methods

### Reagents

**Fig. 1:**
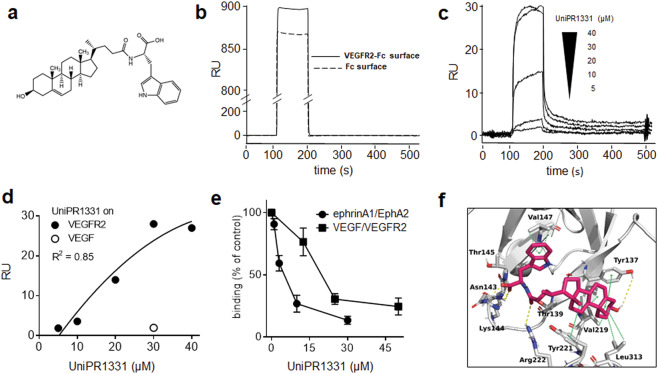
Binding of UniPR1331 to VEGFR2. **a** Chemical structure of UniPR1331**. b** SPR sensorgrams showing the binding of UniPR1331 (40 μM) to the VEGFR2-Fc-coated or to the control Fc-coated surfaces. **c** Blank-subtracted SPR sensorgrams derived from injection of UniPR1331 on the VEGFR2-Fc surface. **d** Steady-state analysis obtained by Scatchard’s plot analysis of the bound RU values at equilibrium from **b**. White dot represents UniPR1331 binding to a control VEGF-coated surface. **e** ELISA-based competition experiments: inhibition curves of the binding of biotinylated ephrin-A1-Fc or VEGF to immobilized EphA2-Fc and VEGFR2 ectodomain by UniPR1331. Data in **b**–**d** are representative of other three experiments that gave similar results. Data in **e** are expressed as percent of binding in respect to control without inhibitor and are the mean ∓ S.E.M. of 3–6 independent experiments. **f** Modeled structure of the UniPR1331/VEGFR2 complex from the final frames of MD showing the key residues involved in the interaction. D2–D3 domain of VEGFR2 is depicted in white cartoons representations. VEGFR2 residues involved in the interaction and UniPR1331 are shown in gray and magenta sticks (oxygen in red, nitrogen in blue). Hydrophilic (H-bonds and salt links) and hydrophobic interactions are indicated with yellow dashed and green dotted lines.

UniPR1331 (3β-hydroxy-Δ^5^-cholenic acid, Fig. 1a) was synthesized as described [22]. VEGFA_165_ (here referred to as VEGF) was provided by K. Ballmer-Hofer (PSI, Villigen, Switzerland). The monomeric recombinant form of the extracellular portion of VEGFR2 fused to Fc was from Immunosource (Zoersel, Belgium). EphA2 and biotinylated ephrin-A1 fused to Fc were from R&D systems (Minneapolis, MN). Human Ig Fc fragment was from Millipore (Bedford, MA). Antiphosphorylated VEGFR2 antibody (#MA5–15170, Y1175), Alexa Fluor 594 anti-mouse IgG, and TNYL-RAW and its scrambled control peptide were from Thermo Scientific (Waltham, MA). Anti-VEGF antibody (#MAB3572) was from R&D systems. Antibody against focal adhesion kinase (FAK; #sc-558), phosphorylated fibroblast growth factor receptor 1 (FGFR1) (#sc-6458, Y766), and VE-cadherin (#sc-6458) were from Santa Cruz Biotechnology (Santa Cruz, CA). Horseradish peroxidase (HRP)-labeled anti-rabbit antibody was from Bio-Rad (Hercules, CA). Streptavidin-HRP, tetramethylbenzidine, and dynasore were from Sigma (St. Louis, MO). Sodium chlorate was from BDH Laboratory Supplies (Pole, UK).

### SPR assay

SPR measurements were performed on a BIAcore X100 instrument and CM5 sensorchips (Cytiva, Marlborough, MA). VEGFR2-Fc or the Fc fragment (used for blank subtraction) (20 μg/ml in 10 mM sodium acetate pH 4.0) was allowed to react with the two flow cells of a CM5 sensorchip preactivated as described [23], leading to the immobilization of 11,614 and 3240 RU (approximately 105 and 154 fmol/mm^2^, respectively). Alternatively, VEGF (5 μg/ml in 10 mM sodium acetate pH 4.5) was immobilized onto a CM5 sensorchip, leading to the immobilization of 9396 RU (~400 fmol/mm^2^). Blank immobilization was performed as control. Increasing concentrations of UniPR1331 in PBS, 0.05% tween 20, 5% DMSO (PBS-DMSO) were injected over the VEGFR2, Fc, or VEGF surfaces for 120 s and then washed until dissociation (500 s). At the end of each run, the surfaces were washed with PBS-DMSO until the complete spontaneous detachment of UniPR1331. No regeneration was required. Binding parameters were calculated as described [24].

### ELISA assay

Ninety six-well ELISA high binding plates (Costar, Corning, NY, USA) were incubated overnight with 100 μl/well of 0.5 µg/ml human VEGFR2 (R&D Systems). Then, plates were washed three times with PBS + 0.05% tween 20, pH 7.5, and blocked by a 1 h incubation at 37 °C with PBS + 1% BSA. After washing, increasing concentrations of UniPR1331 were added and preincubated for 1 h. Then, human biotinylated VEGF-165-Fc (300 ng/ml) was added and incubated for 4 h at 37 °C. After washing, streptavidin-HRP (0.05 μg/ml in PBS + 1% BSA) was added and incubated for 1 h at room temperature. After further washing, stable peroxide buffer pH 5.0 [11.3 g/l citric acid, 9.7 g/l sodium phosphate, 0.02% H_2_O_2_ (30%, m/m, in water)] containing 0.1 mg/ml tetramethylbenzidine was added and the colorimetric reaction was allowed to occur. The reaction was stopped with 3 N HCl and the absorbance at 450 nm was measured using an ELISA plate reader (Sunrise, TECAN, Switzerland). ELISA assays on EphA2 were performed basically as described for VEGFR2. For more details, see Tognolini et al. [25].

### Cell cultures

Human umbilical vein ECs (HUVECs) at passages I–VI were grown on plastic coated with porcine gelatine (Sigma) in M199 medium/20% fetal calf serum (FCS, Gibco Life Technologies, Grand Island, NY), EC growth factor (10 µg/ml), and porcine heparin (Sigma) (100 µg/ml). Fetal bovine aortic endothelial GM7373 cells were transfected to generate stable GM7373-VEGFR2 transfectant cells as described [26]. GM7373 cells were also transfected with a pcDNA3/enhanced yellow fluorescent protein (EYFP) vector harboring the extracellular domain of human VEGFR2 (ECD-VEGFR2) cDNA (provided by K. Ballmer-Hofer, PSI, Villigen, Switzerland) to generate stable ECD-VEGFR2-EYFP GM7373 cells. Parental GM7373 cells and transfectants were grown in Dulbecco’s modified Eagle medium (DMEM, Gibco Life Technologies) + 10% FCS. Murine aortic ECs (MAEc) transfected with mouse VEGFR2 were grown in DMEM 10% FCS. Glycosaminoglycan-deficient A745 CHO-K1 cells [27] were kindly provided by J. D. Esko (University of California, La Jolla, CA), grown in Ham’s F-12 medium/10% FCS, and transfected with the ECD-VEGFR2 cDNA to generate stable ECD-VEGFR2-EYFP A745 CHO-K1 cells [28]. Glioblastoma U251 cells, which express different growth factors including VEGF [29], were grown in DMEM 10% FCS 1 mM sodium pyruvate and nonessential amino acids (Gibco Life Technologies). Cells were stained with DiI cellTracker dyes (Thermo Fisher Scientific) according to the manufacturer’s instructions. Cells were tested regularly for mycoplasma negativity.

### VEGF/VEGFR2 binding assay

To remove high capacity VEGF-binding sites associated to HSPGs whose interaction with VEGF could mask the specific interaction of the growth factor with VEGFR2, cells were treated with chlorate to inhibit sulfation of HS chains as described [28]. Chlorate-treated ECD-VEGFR2-EYFP GM7373 cells were incubated for 90 min at 4 °C with VEGF (75 ng/ml) and UniPR1331 (30 μM) in Hanks’ balanced salt solution (HBSS) with calcium and magnesium, washed with PBS, and fixed in 4% paraformaldehyde. Immunofluorescence analysis was performed using an anti-VEGF antibody and Alexa Fluor 594 anti-mouse IgG. Cells were photographed using a Zeiss Axiovert 200 M epifluorescence microscope (Carl Zeiss, Gottingen, Germany).

### VEGF-mediated cell–cell adhesion assay

CHO-K1 cells expressing HSPGs and devoid of VEGFR2 were seeded in 24-well plates at 5 × 10^4^ cells/cm^2^. After 24 h, ECD-VEGFR2-YFP A745 CHO-K1 cells (5 × 10^4^ cells/cm^2^) were added to CHO-K1 monolayers with VEGF (75 ng/ml) and the indicated concentrations of UniPR1331. After 2 h at 4 °C, ECD-VEGFR2-YFP A745 CHO-K1 cells bound to the CHO-K1 monolayer were photographed and counted.

### Phosphorylation assays

HUVECs, GM7373-VEGFR2, or MAEc-VEGFR2 ECs were starved for 6 h, treated at 37 °C for 10 min with VEGF (10 ng/ml) and UniPR1331 (30 μM), and lysed in lysis buffer (TRIS-HCl pH 7 50 mM, NaCl 150 mM, Triton X100 1%, Brij 0.1%). Then, 40 μg protein/sample was separated by SDS-10% PAGE and analyzed by western blot for the phosphorylated form of VEGFR2, ERK_1/2_ (#4370, Cell Signalling), or PLC-γ (#2821, Cell Signalling). For FGFR1 phosphorylation assay, cells were treated as above with FGF2 at 30 ng/ml.

### VEGFR2 and VE-cadherin internalization

HUVECs were starved, treated with UniPR1331 (30 μM, 20 min) and then with VEGF (30 ng/ml, 30 min at 37 °C), and washed and incubated with biotin-3-sulfo-N-hydroxysuccinimide ester sodium salt (Sigma) (0.5 mg/ml) in HBSS for 1 h at 4 °C. Residual biotin was quenched with 50 mM Tris (pH 8.6) and 100 mM NaCl for 15 min at 4 °C. Cells were washed with cold PBS, lysed in lysis buffer, immunoprecipitated with streptavidin-sepharose (GE-Healthcare), separated on SDS-7.5% PAGE, and analyzed by western blot with anti-VEGFR2 and anti-VE-cadherin antibodies.

### FACs analysis

HUVECs were seeded in six-well plates, grown until 80% of confluence, starved for 2 h, pretreated for 20 min with UniPR1331 or 0.3% DMSO, stimulated with 30 ng/ml VEGF for 30 min, washed with PBS, harvested, and centrifuged. Pellets were suspended in flow cytometry staining buffer and blocked with human IgG1-Fc fragment for 20 min (Millipore). Human VEGFR2-PE-conjugated antibody or mouse IgG-PE-conjugated isotype control antibody (R&D systems) was added and incubated at 25 °C. Unbound antibody was removed by washing and VEGFR2 internalization was revealed by flow cytometry analysis (Guava easyCyte 5, Millipore). Data were analyzed with FlowJo software (Ashland, OR).

### Immunofluorescence analysis for VE-cadherin

Confluent GM7373-VEGFR2 cells seeded on µ-slide eight-well chambers (IBIDI, Gräfelfing, Germany) were treated with UniPR1331 (30 µM) for 20 min and then with VEGF (30 ng/ml) for 45 min, fixed in 4% paraformaldehyde in PBS, permeabilized with 0.2% Triton-X100, and saturated with 3% BSA in PBS. Then, cells were incubated with anti-VE-cadherin antibody. Nuclei were counterstained with DAPI. Cells were photographed using a Zeiss Axiovert 200 M epifluorescence microscope equipped with Apotome and a Plan-Apochromat ×63/1.4 NA oil objective.

### Permeability assay

GM7373-VEGFR2 cells (2 × 10^5^) were plated in Transwell chambers (Costar, 6.5 mm diameter, 3.5 μm pore size), grown for 3 days, starved for 2 h, treated with UniPR1331 (30 µM) for 20 min, and stimulated with VEGF (30 ng/ml) for 45 min. Biotinylated-BSA (4 μg/ml) was added to the upper chamber for 2 h. The amount of biotinylated-BSA in the lower chamber was determined by ELISA with streptavidin-HRP and colorimetric read at 405 nm (microplate reader ELx800, Labtek).

### Proliferation assay

HUVECs were seeded at 17,500 cells/cm^2^ onto tissue culture 48-well plates in M199 + 2.5% FCS. The following day cells were incubated for 24 or 48 h at 37 °C with VEGF (30 ng/ml) and the indicated concentrations of UniPR1331 and then counted using the MACSQuant Analyzer (Miltenyi Biotec, Bologna, Italy).

### Wound monolayer assay

Confluent cultures of HUVECs were starved, wounded with a rubber policeman, and incubated for 24 h at 37 °C with VEGF (30 ng/ml) and the indicated concentrations of UniPR1331. Then, the extent of wound repair was evaluated by measuring the area of the wound by computerized image analysis using the ImageJ software (http://rsb.info.nih.gov/ij/).

### EC sprouting assay

Sprouting of HUVEC aggregates (spheroids) embedded in fibrin gel was analyzed as described [26].

### Zebrafish yolk membrane angiogenesis assay

Zebrafish (*Danio rerio*) adults (wild-type AB strain or transgenic Tg(*fli*1:EGFP)^y1^) were maintained at 28 °C on a 14 h light/10 h dark cycle under standard laboratory conditions [30]. Embryos at 48 h post fertilization were anesthetized with 0.016% Tricaine (Sigma) and injected into the perivitelline space with U251 cells (400–600 cells/embryo), followed by a second injection with 0.5% DMSO alone or containing 50 μM UniPR1331 in the proximity of subintestinal vein (SIV) vessels using an InjectMan IN2 microinjector (Eppendorf, Milan, Italy) equipped with FemtoJet. The angiogenic response was evaluated at 72 h post fertilization after alkaline phosphatase (AP) staining [31]. Images of embryo’s SIV were acquired using an Axio Zoom.V16 fluorescence microscope (Carl Zeiss) equipped with a digital camera. The following parameters were evaluated: total number and total length (express in μm) of ectopic AP + vessels sprouting from the SIVs on both sides of the embryo body. For light sheet images, embryos were anesthetized in fish water containing 0.016% Tricaine and embedded within glass capillary filled with low melting agarose (1% low melting agarose:fish water; 1:1). The Lightsheet Z.1 fluorescence microscope (Carl Zeiss) imaging chamber was filled with fish water and Tricaine and maintained at 33 °C. Three-dimensional images of z-stack files were reconstructed with arivis Vision4D software (arivis AG, Munich, Germany).

### Statistical analysis

Statistical analyses were performed using the statistical package Prism 6. Student’s *t*-test or one-way ANOVA analysis of variance followed by Bonferroni multiple comparison post-test were performed. Data were expressed as mean ± SEM. Differences were considered significant when *P* < 0.05.

## Results

### UniPR1331 interacts with VEGFR2 in the D2 domain and the D2–D3 hinge region

The capacity of cholenic acid derivative UniPR1331 (Fig. 1a) to interact with VEGFR2 was assessed by SPR. As shown in Fig. 1b, c, UniPR1331 binds VEGFR2 in a specific and dose-dependent way with an almost complete spontaneous detachment at the end of the injection. The Kd value of the interaction, calculated by Scatchard’s plot analysis (Fig. 1d), is 62.2 ± 2.1 μM (from three independent determinations), thus significantly higher than that determined under the same experimental conditions for the UniPR1331/EphA2 interaction (3.3 μM) [22]. Important to note, SPR analyses also demonstrated that UniPR1331 does not interact directly with the VEGFR2 natural ligand VEGF (Fig. 1d).

We then evaluated if, by binding to VEGFR2, UniPR1331 prevents VEGF interaction by using the ELISA assay already used to evaluate the capacity of PPI-is to disrupt the ephrin-A1/EphA2 receptor interaction ﻿[22]. As shown in Fig. 1e, UniPR1331 prevents the binding of VEGF to VEGFR2 in a dose-dependent way with an IC_50_ equal to 16 μM. In the same experimental conditions, UniPR1331 prevents the binding of ephrin-A1 to EphA2 with higher potency (IC_50_ = 4 μM), mirroring the Kd values measured by SPR.

The ability of UniPR1331 to compete with VEGF for binding to the receptor indicates that UniPR1331 interacts with the ligand-binding domain of VEGFR2 located in the D2–D3 domain [32]. To obtain molecular insight into how UniPR1331 binds to VEGFR2, we exploited molecular modeling and simulation, by docking UniPR1331 to the crystallographic structure of the D2–D3 domain construct. In the best selected docking pose (Gscore, −5.2 kcal/mol), UniPR1331 is stabilized by hydrophobic interactions between its steroid core and Thr139, Val219, Tyr221, and Leu313 of VEGFR2 (data not shown), underscoring the importance of lipophilicity in the binding.

To assess its stability, the docked VEGFR2–UniPR1331 complex was subjected to MD simulations. Three replica simulations (50 ns duration) were generated that showed convergence to a structure in which UniPR1331 induced a rotation on the D2–D3 hinge region with a 45° clockwise shift of the D2 domain with respect to the D3 domain, causing the loss of the H-bonds between Asn140, Arg220 and Glu251 with consequences in determining the orientation of the D2–D3 region and the creation of the VEGF-binding pocket. The hydrophobic interactions identified during docking were maintained during the MD simulations with additional interactions: the 3β-hydroxyl group of the steroid core was stabilized by an H-bond interaction with Tyr137, which also made a hydrophobic interaction with ring A of the steroid. The amide group (which connects the steroid core and the L-Trp moiety of UniPR1331) was stabilized by H-bonds with Thr139 and Arg222. The free carboxyl group instead formed a complex H-bond network that involved Asn143, Lys144, and Thr145. Finally, the orientation of the indole ring was maintained through hydrophobic interactions with Val147 (Fig. 1f and Fig. S1). All the replicas showed that the D2–D3 structure was well maintained (RMSD of the corresponding residues less than 3 Å from the starting structure), as was that of UniPR1331 (RMSD less than 1 Å) (Fig. S2), indicating that the proposed binding of UniPR1331 is rather stable.

We then searched for structural similarities shared by UniPR1331 and dimeric VEGF responsible for their capacity to bind the D2–D3 domain of VEGFR2. Visual inspection of the two systems showed that the ring A composing the steroid core of UniPR1331 overlaps the hydrophobic interface of VEGF, where Ile46 should pack against Val217 in D2–D3 linker region, thus simulating the same hydrophobic interaction. Also, the polar core of UniPR1331 packs against Arg222 in the D2–D3 linker region, where VEGF should place Glu64. Finally, methyl groups of UniPR1331 points against strand β5 of VEGF where Pro85 should pack against Gly255 in D3. Overall, we can assess both hydrophobic and hydrophilic similarities between the binding modes of UniPR1331 and VEGF to VEGFR2 (Fig. S3).

In conclusion, UniPR1331 mimics VEGF, being able to bind and mask its binding pockets on VEGFR2. Moreover, upon binding, UniPR1331 induces an allosteric conformational drift on the hinge region of VEGFR2 that contributes to bury the VEGF-binding site, providing a basis for further optimization of the inhibitory activity of UniPR133.

### Effect of UniPR1331 on the VEGF/VEGFR2 interaction at the EC surface

**Fig. 2:**
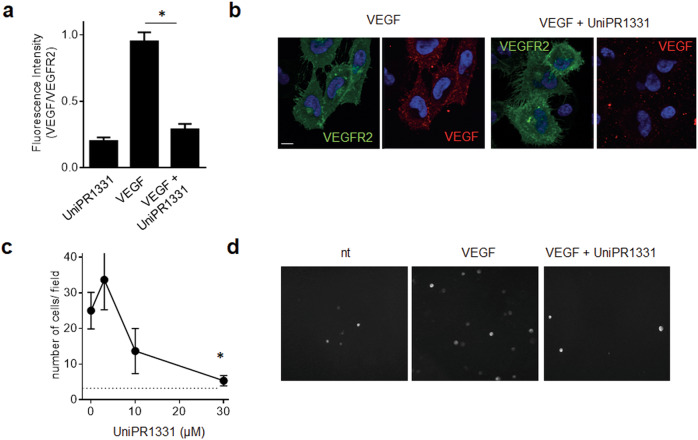
Effect of UniPR1331 on VEGF/VEGFR2 binding at the cell surface. **a** Chlorate-treated ECD-VEGFR2-EYFP GM7373 cells were incubated with VEGF and UniPR1331 and analyzed in immunofluorescence with anti-VEGF antibody. Fluorescence was quantified using ImageJ software as corrected total cell fluorescence: integrated density − (area of selected cell × mean fluorescence of background). Data are the mean ± S.E.M. of measurements on 25–35 cells for each sample from two independent experiments. (*P < 0.0001 in respect to cell treated with VEGF alone). **b** Microphotographs of ECD-VEGFR2-YFP GM7373 incubated with VEGF and UniPR1331. Scale bar: 10 μM. **c** ECD-VEGFR2-YFP A745 CHO cells were added to HSPG-bearing CHO-K1 monolayers with VEGF and UniPR1331. Adherent cells were photographed and counted after 2 h of incubation. Data are the mean ± S.E.M. of cell count in six microscopic fields. **d** Microphotographs of ECD-VEGFR2-YFP A745 CHO-K1 incubated on HSPG-bearing CHO-K1 monolayers with VEGF and UniPR1331.

We then studied the effect of UniPR1331 at the EC surface exploiting the VEGF/VEGFR2 binding assay model with chlorate-treated ECD-VEGFR2-EYFP GM7373. As shown in Fig. 2a, b, VEGF colocalizes with EYFP-VEGFR2 at the cell surface and this association is prevented by UniPR1331, indicating that the compound binds and masks VEGFR2 to VEGF also on living cells.

HSPGs mediate the engagement of VEGF with VEGFR2 with the formation of the HSPGs/VEGF/VEGFR2 ternary complexes [33]. To evaluate the capacity of UniPR1331 to prevent the formation of this complex, we exploited the cell–cell adhesion assay in which, due to its capacity to bind simultaneously to VEGFR2 and HSPGs expressed in trans on neighboring cells, VEGF mediates cell–cell adhesion [34]. As shown in Fig. 2c, d, VEGF can bind simultaneously to HSPGs expressed by a monolayer of CHO-K1 cells and to VEGFR2 expressed on the surface of ECD-VEGFR2-YFP A745 CHO-K1 cells in suspension, allowing the substrate adhesion of the latter. When added, UniPR1331 prevents in a dose-dependent way the adhesion of ECD-VEGFR2-YFP A745 CHO-K1 cells, further confirming that UniPR1331 retains its capacity to bind VEGFR2 at the cell surface, preventing the formation of the HSPGs/VEGF/VEGFR2 complex.

### Effect of UniPR1331 on VEGFR2 activation and signal transduction in ECs

**Fig. 3:**
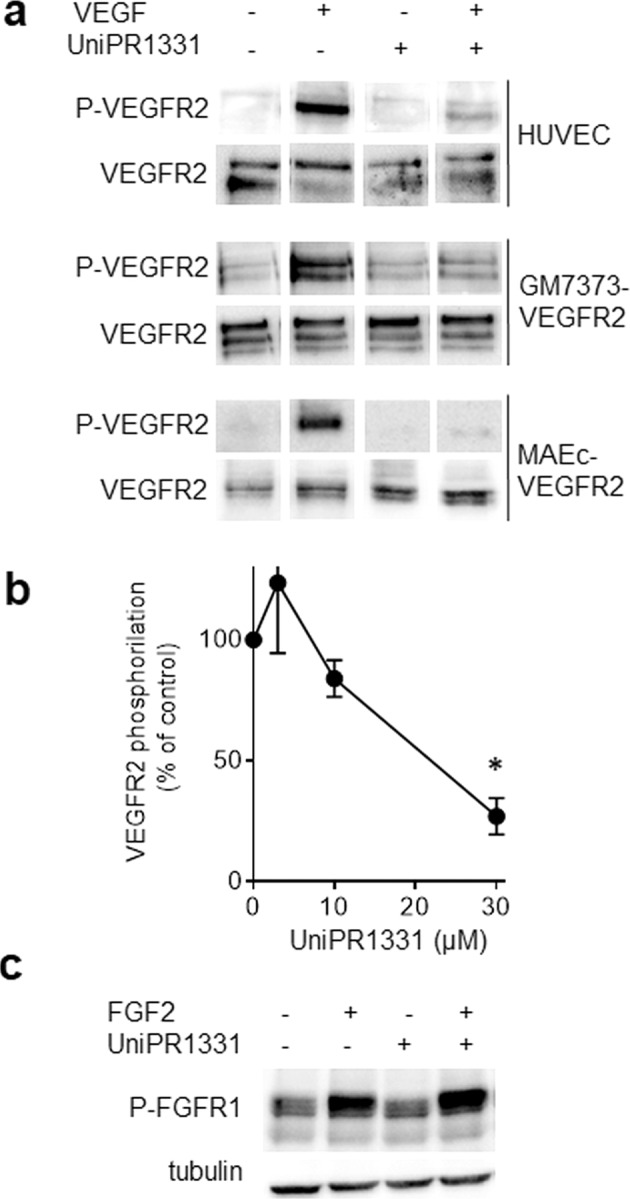
Effect of UniPR1331 on VEGFR2 activation. The indicated cells were left unstimulated or stimulated with 10 ng/ml VEGF (**a**, **b**) or 30 ng/ml FGF2 (**c**) with UniPR1331 (30 μM in **a**–**c** or increasing concentrations in **b**). Then, cells were analyzed by WB with anti-P-VEGFR2 (**a**, **b**) or anti-P-FGFR1 (**c**) antibody. Uniform loading was confirmed with anti-VEGFR-2 or anti-tubulin antibody. The results shown are representative of other two that gave similar results. The single lanes have been cropped and reorganized for an easy comparison. **b** Densitometric quantification of P-VEGFR2 immunoreactive bands normalized to the expression levels of VEGFR2. (mean ± S.E.M. of three independent experiments, *P < 0.005).

VEGF engagement causes VEGFR2 homodimerization, autophosphorylation, and internalization that trigger a complex signal transduction leading to ECs proangiogenic activation [2, 35]. UniPR1331 inhibits VEGF-dependent autophosphorylation of VEGFR2 in different EC lines and in HUVECs in a dose-dependent way with an ID_50_ equal to 22 μM (see Fig. 3a, b and Fig. S4 for original uncropped blots). The specificity of the inhibitory effect of UniPR1331 was evaluated on another RTK, namely the FGFR1. As shown in Fig. 3c, UniPR1331 does not affect the activation of this receptor by its natural ligand FGF2, suggesting that the effect of UniPR1331 on VEGFR2 is specific despite its relatively low affinity binding.

**Fig. 4:**
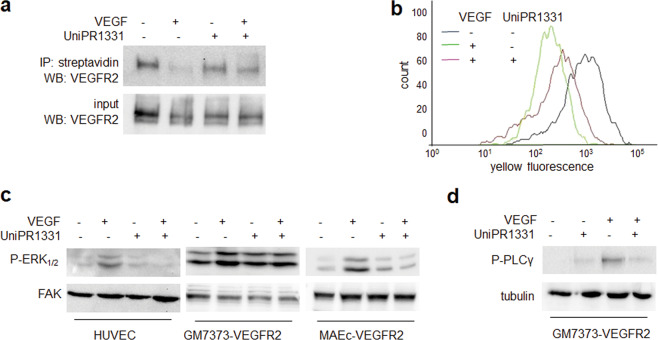
Effect of UniPR1331 on VEGFR2 internalization and signal transduction. HUVECs unstimulated or stimulated with VEGF and UniPR1331 were analyzed for VEGFR2 internalization by WB (**a**) or by FACs analysis (**b**) or for the activation of VEGF-dependent signal transduction by WB with anti-P-ERK_1/2_ (**c**) or anti-P-PLC-γ (**d**) antibody. Uniform loading was confirmed by WB with anti-FAK (**c**) or anti-tubulin (**d**) antibody. The results shown are representative of two to three other experiments that gave similar results.

Since VEGFR2 is endocytosed by ECs when engaged by VEGF [36], we evaluated the effect of UniPR1331 on VEGFR2 internalization. As shown by both surface biotinylation assay (Fig. 4a) and FACs analysis (Fig. 4b), the amount of cell surface-associated VEGFR2 is reduced in cells stimulated with VEGF but not to the same extent in those exposed also to UniPR1331, indicating that, by hampering VEGFR2 engagement by VEGF, it inhibits the internalization of the complex.

VEGF/VEGFR2 endocytosis is essential for ERK_1/2_ phosphorylation [35, 36], which is in turn required for EC proangiogenic activation [2]. Also, ERK_1/2_ is phosphorylated upon EphA2 activation [37, 38], suggesting that it represents a point of convergence for the VEGF/VEGFR2 and EphA2/ephrin-A1 systems. As shown in Fig. 4c, UniPR1331 inhibits, with different potency, the phosphorylation of ERK_1/2_ in HUVECs, and other different EC lines (see Fig. S5 for original uncropped blots). Also PLC-γ activation is triggered by VEGF-dependent VEGFR2 activation and internalization and is implicated in proangiogenic activation and vascular permeability [39, 40]. Again, we found that UniPR1331 exerts a significant inhibitory effect on the VEGF-dependent VEGFR2 activation of this second messenger too (Fig. 4d).

### Effect of UniPR1331 on VEGF-dependent endothelial permeability

**Fig. 5:**
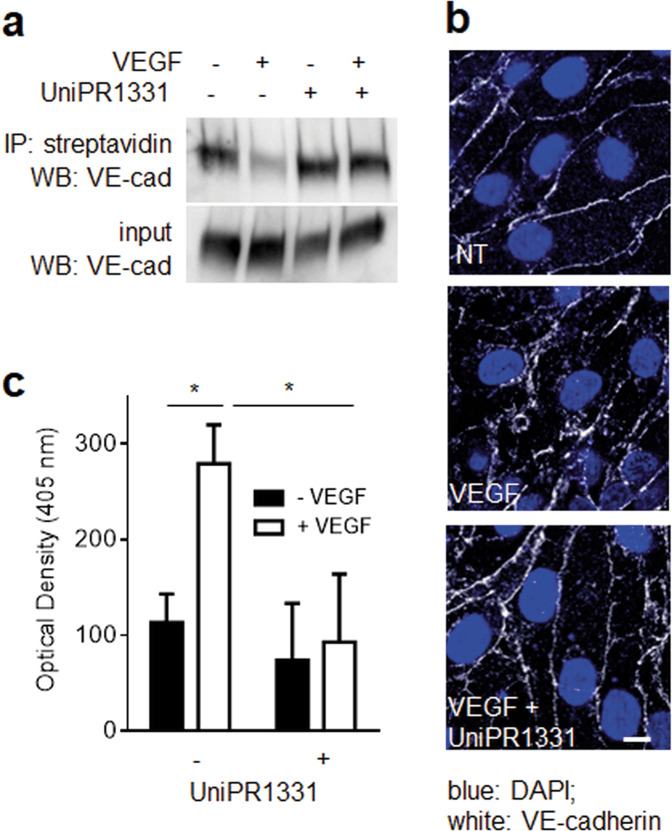
Effect of UniPR1331 on VEGF-induced VE-cadherin internalization and permeabilization in ECs. **a** HUVECs unstimulated or stimulated with VEGF and UniPR1331 were analyzed for VE-cadherin internalization by WB. **b** Microphotographs of GM7373-VEGFR2 cells incubated with VEGF and UniPR1331 and immunostained for VE-cadherin (scale bar: 10 μm). **c** Monolayer of GM7373-VEGFR2 cells unstimulated or stimulated with VEGF and UniPR1331 were evaluated for permeability as described in “Material and methods.” The results shown are the mean ± S.E.M. of three independent experiments; *P < 0.05.

VEGF stimulation promotes the rapid endocytosis of the adhesion molecule VE-cadherin, which plays a pivotal role in regulating the endothelial barrier and the vascular leakage associated with many human diseases [40, 41]. In normal conditions, VE-cadherin is mainly located at the EC membrane and VEGF stimulation promotes its internalization [42] and redistribution inside the cells (Fig. 5). UniPR1331, by masking VEGFR2 to VEGF, hampers VE-cadherin internalization and effectively prevents endothelial permeability in an in vitro model of vascular leakage (Fig. 5).

### Effect of UniPR1331 on VEGF-dependent proangiogenic activation of ECs

**Fig. 6:**
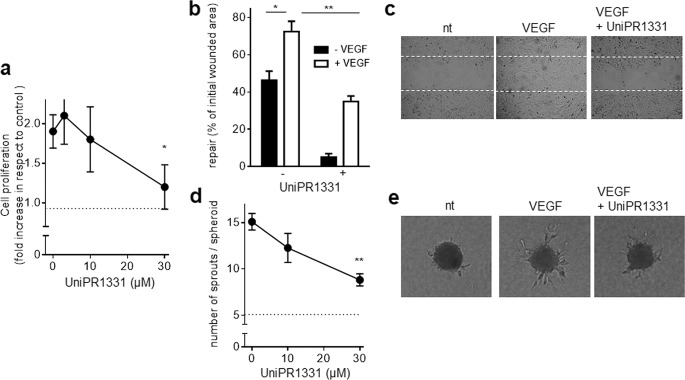
Effect of UniPR1331 on VEGF/VEGFR2-dependent EC proangiogenic activation. **a** HUVECs stimulated with VEGF and increasing concentrations of UniPR1331 were counted using the MACSQuant Analyzer. Data are expressed as proliferation fold increase in respect to HUVECs cells left untreated (dashed line). **b** HUVEC monolayers were wounded and incubated with VEGF and UniPR1331. Then, the extension of the repaired wound area was evaluated. Data are expressed as percent or repaired area in respect to the extension of the original wound. **c** Microphotographs of wounded HUVEC monolayers taken after 24 h of incubation with or without VEGF and UniPR1331. Dashed lines mark the edge of the wound at *t*_0_. **d** HUVEC spheroids embedded in fibrin gel were incubated with VEGF and UniPR1331. Then, radially growing cell sprouts were counted. **e** Microphotographs of spheroids incubated with VEGF and UniPR1331. Data shown in **a**, **b**, and **d** are the mean ± S.E.M. of three independent experiments (*P < 0.05 and **P < 0.01).

VEGF-dependent VEGFR2 activation and consequent signal transduction induce EC proangiogenic activation consisting of an increased proliferation, migration, and invasive capacity [43]. As shown in Fig. 6a, VEGF induces a twofold increase of HUVECs proliferation that is inhibited by UniPR1331 in a dose-dependent way. The effect cannot be ascribed to an aspecific cytotoxic effect since UniPR1331 does not induce release of LDH under the same experimental conditions in which it instead significantly inhibits VEGF-induced cell proliferation (Fig. S6).

Motogenic activity consists in a regenerative potential that allows the repair of a mechanically wounded EC monolayer as the result of the capacity of VEGF to induce EC proliferation and motility [44]. UniPR1331 inhibits VEGF motogenic activity in wounded HUVEC monolayers (Fig. 6b, c). Finally, UniPR1331 also retains its inhibitory activity in an in vitro three-dimensional model of angiogenesis in which EC spheroids stimulated by VEGF invade a fibrin matrix, generating endothelial sprouts as a result of the localized breakdown of the extracellular matrix that occurs together with EC migration and growth [45] (Fig. 6d, e).

### Effect of UniPR1331 on tumor-driven angiogenesis in vivo

**Fig. 7:**
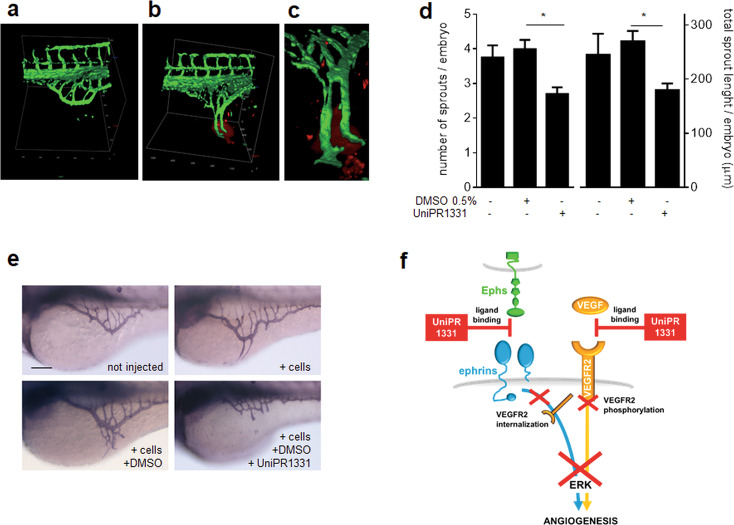
Effect of UniPR1331 on in vivo angiogenesis. 3D images reconstruction of SIV in Tg(*fli*1:EGFP)^y1^ zebrafish embryos in the absence (**a**) or in the presence (**b**) of U251 cells (in red). **c** High magnification of the image reconstruction of **b** showing the detail of ectopic SIV vessels converging toward U251 cells. **d** Evaluation of the angiogenic response: quantification of AP + ectopic sprouts are expressed as number of sprouts/embryo and cumulative length/embryo normalized in respect to noninjected embryos (number of embryos analyzed: U251 alone, 32; U251 + DMSO, 51; U251 + UniPR1331, 47). Data are the mean ± S.E.M. of three independent experiments (*P < 0.05). **e** Representative images of the angiogenic response used for the quantifications reported in **d**. Scale bar: 100 µm. **f** Schematic representation of the multitarget mechanisms of action of UniPR1331. By binding to Ephs, UniPR1331 inhibits VEGFR2 internalization required for downstream signaling. Simultaneously, UniPR1331 binds VEGFR2, hampering its interaction with VEGF and receptor phosphorylation. Both these inhibitions contribute to prevent phosphorylation of ERK_1/2_, a key second messenger for EC proangiogenic activation.

The antiangiogenic activity of UniPR1331 was assessed in vivo in the zebrafish yolk membrane angiogenesis assay using U251 glioblastoma cells as angiogenic stimulus, thus mimicking the actual process of tumor neovascularization [46]. Injection of UniPR1331 or DMSO alone did not exert any effect on physiological SIV development (Fig. S7). U251 cells effectively induce angiogenesis, causing the sprouting of SIV that converge toward implanted cells in zebrafish embryos 24 h after injection (Fig. 7a–c). This angiogenic response is inhibited by UniPR1331 both in terms of number of ectopic sprouts and of their total length when injected along with cells (Fig. 7d, e).

## Discussion

An enormous effort is being devoted to devising efficacious anticancer therapies. Among these, those directly aimed at inhibiting tumor cell proliferation and dissemination are usually targeted to specific structures (i.e., receptors) expressed specifically and/or ex novo on tumor cells. In this regard, Ephs RTKs and their membrane-bound ephrin ligands are classically overexpressed on tumor cells [7], thus presenting an ideal target. Accordingly, anti-Eph strategies have been developed [47], some of which are currently under clinical trial (https://clinicaltrials.gov/ct2/results?cond=&term=ephrin&cntry=&state=&city=&dist=).

A challenging alternative is to block cancer growth by preventing its neovascularization. Accordingly, the VEGF/VEGFR2 system has been the object of intense study, leading to the development of the anti-VEGF monoclonal antibody bevacizumab, currently employed in cancer therapy. Unfortunately, all the antiangiogenic drugs developed so far have provided limited clinical benefits when used in a monotherapy regimen, likely due to the development of drug resistance and the biochemical redundancy of both the cancerogenesis and angiogenesis processes [15, 48]. This stalemate has been tentatively overcome by identifying combinations of drugs specifically directed against different molecular targets [49] or by designing multitarget drugs able to block multiple therapeutic targets simultaneously.

Interestingly, VEGFR2 and Ephs represent two promising objectives for the development of multitarget drugs, as they are expressed in both cancer and ECs and are involved in tumor cell proliferation, metastatization [50–52], and neovascularization [4–6].

Also, a cross talk exists between the VEGF/VEGFR2 and Eph/ephrin systems: ephrin-B2 physically associates with VEGFR2 to form a complex stabilized by syntenin and is required for VEGFR2 activation [6]. Also, EphB4 interferes with VEGFR2 downstream signaling leaving VEGF-dependent VEGFR2 activation unaffected [53]. Finally, EphB4 and ephrin-B2 are required for VEGFR2 internalization and downstream signaling [4, 54].

Taken together, these observations suggest the possibility that UniPR1331 may inhibit VEGF-dependent angiogenesis merely as a consequence of the inhibition of the Eph/ephrin system, a possibility further supported by the fact that UniPR1331 is less potent on VEGFR2 than on EphA2 (Fig. 1). To evaluate this possibility, we performed VEGFR2 phosphorylation assays in the presence of TNYL-RAW (that blocks ephrin-B2/EphB4 interaction [55]) and of dynasore (a known inhibitor of VEGFR2 internalization [56] that, in turn, is required to induce a full angiogenic response and is mediated by the EphB4/ephrin-B2 system [4, 54]). As shown in supplementary Fig. S8, TNYL-RAW and dynasore do not inhibit VEGF-dependent phosphorylation of VEGR2, suggesting that UniPR1331 exerts its inhibitory potential that is, at least in part, independent of the Eph/ephrin system.

Regarding its mechanism of action, UniPR1331 does not inhibit receptor kinase activities by acting intracellularly. Indeed, it does not physically interact with the intracellular kinase domains of EphA2 and VEGFR2 in a LANCE assay, leaving their enzymatic activity unaffected [14, 22]. Conversely, here we demonstrated that UniPR1331 physically interacts with the extracellular portion of VEGFR2. Accordingly, computational studies indicate that UniPR1331 acts as a VEGF mimicking compound, by interacting and masking the VEGF-binding domain of VEGFR2 and by inducing a conformational drift of VEGFR2 that further buries the VEGF-binding site. Taken together, these observations point to UniPR1331 as a PPI-i of VEGFR2 that, in respect to intracellularly acting compounds (i.e., pan-kinase inhibitors), whose efficiency suffers from cell internalization and delivery to the intracellular microenvironment [57], may have great therapeutic potential as a new class of multitarget anticancer drugs [58]. In effect, UniPR1331 seems to act simultaneously as a genuine VEGFR2-PPI-i (inhibiting VEGF-dependent receptor phosphorylation) and as a pan-Eph PPI-i (inhibiting VEGFR2 internalization), with a cumulative effect on downstream signaling (i.e., ERK_1/2_ activation) (Fig. 7f) that could be advantageous in respect to classic monotherapies, which are instead prone to the development of drug resistance. The multitarget activity of UniPR1331 possibly contributes to its robust antiangiogenic potential that is retained in vivo in the zebrafish yolk membrane in which angiogenesis is driven by VEGF-releasing tumor cells [46] (Fig. 7d, e). This model has already been demonstrated to be highly predictive when employed to evaluate compounds for their antiangiogenic potential. Accordingly, also by means of more complex, expensive and time-consuming U251 and U87MG mouse xenografts, UniPR1331 has been demonstrated to exert an antiangiogenic effect with an efficiency that is slightly higher than that of bevacizumab [14].

Interestingly, bevacizumab has failed to demonstrate an overall survival benefit in two large phase III randomized trials, and is currently under investigations aimed at evaluating combinatorial therapies with reirradiation and immunotherapy [59]. It would be interesting to evaluate such combinations also for the multitarget UniPR1331.

Besides neovascularization, UniPR1331 inhibits VEGF-induced vascular permeability, which is an important aspect of tumor angiogenesis and consequent metastatization. Importantly, an abnormal vascular permeability is also involved in other pathologies including diabetic retinopathy [60], neurological disorders [61], and lung injury [62] and, interestingly, besides the VEGF/VEGFR2 system [63, 64], the Eph/ephrin system is also involved in the regulation of vascular permeability [6, 65, 66]. Accordingly, the effect of inhibitors of the Eph/ephrin system has been evaluated in diabetes [19] and Alzheimer [67].

## Supplementary information

Supplemental material
