# Correction: Cholenic acid derivative UniPR1331 impairs tumor angiogenesis via blockade of VEGF/VEGFR2 in addition to Eph/ephrin

**DOI:** 10.1038/s41417-021-00391-9

**Published:** 2021-09-30

**Authors:** Marco Rusnati, Giulia Paiardi, Chiara Tobia, Chiara Urbinati, Alessio Lodola, Pasqualina D’Ursi, Miriam Corrado, Riccardo Castelli, Rebecca C. Wade, Massimiliano Tognolini, Paola Chiodelli

**Affiliations:** 1 Experimental Oncology and Immunology, Department of Molecular and Translational Medicine, University of Brescia, Brescia, Italy; 2 Molecular and Cellular Modeling Group, Heidelberg Institute for Theoretical Studies, Heidelberg, Germany; 3 Department of Food and Drug, University of Parma, Parma, Italy; 4 Institute for Biomedical Technologies, National Research Council (ITB-CNR), Segrate, MI Italy; 5 Center for Molecular Biology (ZMBH), DKFZ-ZMBH Alliance, Heidelberg University, Heidelberg, Germany; 6 Interdisciplinary Center for Scientific Computing (IWR), Heidelberg University, Heidelberg, Germany

**Keywords:** Cell biology, Tumour angiogenesis

Correction to: *Cancer Gene Therapy* 10.1038/s41417-021-00379-5, published online 23 August 2021

The article “Cholenic acid derivative UniPR1331 impairs tumor angiogenesis via blockade of VEGF/VEGFR2 in addition to Eph/ephrin”, written by Marco Rusnati, Giulia Paiardi, Chiara Tobia, Chiara Urbinati, Alessio Lodola, Pasqualina D’Ursi, Miriam Corrado, Riccardo Castelli, Rebecca C. Wade, Massimiliano Tognolini and Paola Chiodelli, was originally published electronically on the publisher’s internet portal on 23 August 2021 without open access. With the author(s)’ decision to opt for Open Choice the copyright of the article changed on 16 September 2021 to © The Authors 2021 and the article is forthwith distributed under a Creative Commons Attribution 4.0 International License, which permits use, sharing, adaptation, distribution and reproduction in any medium or format, as long as you give appropriate credit to the original author(s) and the source, provide a link to the Creative Commons licence, and indicate if changes were made. The images or other third party material in this article are included in the article’s Creative Commons licence, unless indicated otherwise in a credit line to the material. If material is not included in the article’s Creative Commons licence and your intended use is not permitted by statutory regulation or exceeds the permitted use, you will need to obtain permission directly from the copyright holder. To view a copy of this licence, visit http://creativecommons.org/licenses/by/4.0.

