# Supplementary material for: Cholenic acid derivative UniPR1331 impairs tumor angiogenesis via blockade of VEGF/VEGFR2 in addition to Eph/ephrin
Source: Cancer Gene Ther. 2021 Aug 23;29(7):908–17. doi: 10.1038/s41417-021-00379-5 (PMC9293752; doi:10.1038/s41417-021-00379-5)

Cholenic acid derivative UniPR1331 impairs tumor angiogenesis via blockade of VEGF/VEGFR2 in addition to Eph/ephrin

Marco Rusnati, Giulia Paiardi, Chiara Tobia, Chiara Urbinati, Alessio Lodola, Pasqualina D'Ursi, Miriam Corrado, Riccardo Castelli, Rebecca C Wade, Massimiliano Tognolini and Paola Chiodelli

Supplementary material and methods

LDH assay: HUVECs were seeded in 96-well plates at a density of 10^5 cells/ml and the day after treated with UniPR1331 for 2 or 16 h at 37°C. Then, released LDH was measured by the CytoTox 96 assay following manufacturer's protocol (Promega, Madison, WI). Using an ELISA plate reader (Sunrise, Tecan) at 492 nm.

Computational studies: The structure of the D2-D3 region of VEGFR2 was obtained from the crystal structure of RCSB-PDB ID: 2X1X (1) and refined by adding missing residues 206-207 by homology modelling using the SwissModel web-server (https://swissmodel.expasy.org/). Subsequently, the model was protonated using PROPKA at pH7 with the Protein Preparation Wizard Tool (Schrodinger Suite, Protein Preparation Wizard; Epik version 2.6. Impact version 6.1; Prime version 3.4, Schrodinger, LLC, New York, NY, 2013). With the same tool, the protein structure was prepared for docking calculations and submitted to restrained energy minimization using the OPLS 2005 force field to an RMSD of 0.3 Å for non-hydrogen atoms. The structure of UniPR1331 (2) was prepared for docking calculations using LigPrep by considering tautomeric and ionization states, ring conformations and stereoisomers, and then energy minimized using MacroModel with the OPLS 2005 force field to an energy gradient of 0.05 kJ/(mol Å).
Docking simulations: were performed with Glide 4.8 (Glide Schrödinger Release 2019-4: Glide, Schrödinger, LLC, New York, NY, 2019) in Standard Precision mode with default parameters. The grid box for the docking of 80 Å x 80 Å x 80 Å was centred on the hinge region between D2 and D3, comprising residue Ile215 to Tyr221, Tyr165, Phe166, Ser193, Tyr194, Ala195, Gly196, Met197, Leu252, Asn253, Val254, Gly255, Ile256, Asp257, Phe258, Val273, Asn274, Arg275, Asp276, Lys286, Phe288, Ser311, Gly312, Leu313. Thirty-two poses were collected and subsequently ranked according to their Gscore value. The pose selected was the top ranked with all the junctions between the rings of the UniPR1331 steroid core in trans position.
Energy Minimization and Molecular Dynamics (MD) simulation: The Amber18 package (3) was used to run all-atom MD simulations in explicit solvent of D2-D3-VEGFR2 in the presence of UniPR1331.

Three replicas of the system were generated starting from the selected docking pose of UniPR1331. Parameters were assigned using the LEaP program with the ff14SB force field for the protein and Gaff for the compound. Each system was placed in a periodic box of water molecules using the TIP3PBOX water model with 10 Å buffer between the protein and the box edge and then neutralized with counter ions. The simulated systems were firstly energy minimized in 4 steps with decreasing restraints of 10, 5, 1 and 0 kcal/mol $Å^2$ to remove possible bad contacts from the initial structure on the protein and a cut-off for the non-bonded interactions of 8 Å. The systems were then heated in two steps (from 10-100° and from 100-310 °K) using a Langevin thermostat to maintain the systems in the NVT-ensemble. Following this, the systems were then equilibrated for 4 ns without positional restraints in the NPT ensemble before carrying out production simulation of 50 ns duration. The simulations in the NPT ensemble were performed using a Langevin thermostat with a collision frequency of 2 ps^{-1} for temperature regulation. A non-bonded interaction cut-off of 8 Å was employed and the electrostatic interactions were computed by using the particle mesh Ewald algorithm. Covalent bonds involving hydrogen were constrained with the SHAKE algorithm, allowing an integration time step of 2 fs. Analysis of MD simulation and interaction fingerprint Analysis results were carried out using the Cpptraj package (3) and MD-IFP (4).

References

1. Leppanen VM, Prota AE, Jeltsch M, Anisimov A, Kalkkinen N, Strandin T, et al. Structural determinants of growth factor binding and specificity by VEGF receptor 2. Proceedings of the National Academy of Sciences of the United States of America. 2010;107(6):2425-30. Epub 2010/02/11.
2. Castelli R, Tognolini M, Vacondio F, Incerti M, Pala D, Callegari D, et al. Delta(5)-Cholenoyl-amino acids as selective and orally available antagonists of the Eph-ephrin system. European journal of medicinal chemistry. 2015;103:312-24. Epub 2015/09/14.
3. DA C, K B, IY B-S, SR B, TEI C, VWD C, et al. AMBER 2020, University of California, San Francisco. 2020.
4. Kokh DB, Kaufmann T, Kister B, Wade RC. Machine Learning Analysis of tauRAMD Trajectories to Decipher Molecular Determinants of Drug-Target Residence Times. Frontiers in molecular biosciences. 2019;6:36. Epub 2019/06/11.

Supplementary figures

Supplementary Fig. S1 Interaction fingerprint analysis calculated for each of the three replicas along the MD simulations. Each plot shows the development of the detected Interaction fingerprint between UniPR1331 and VEGFR2-D2-D3 residues over the 50 ns duration of the trajectory (one every hundred frames) from top to bottom. HY: hydrophobic interaction; HA: H-bond acceptor: HD: H-bond donor. Blue indicates the presence of an interaction while yellow indicates its absence.

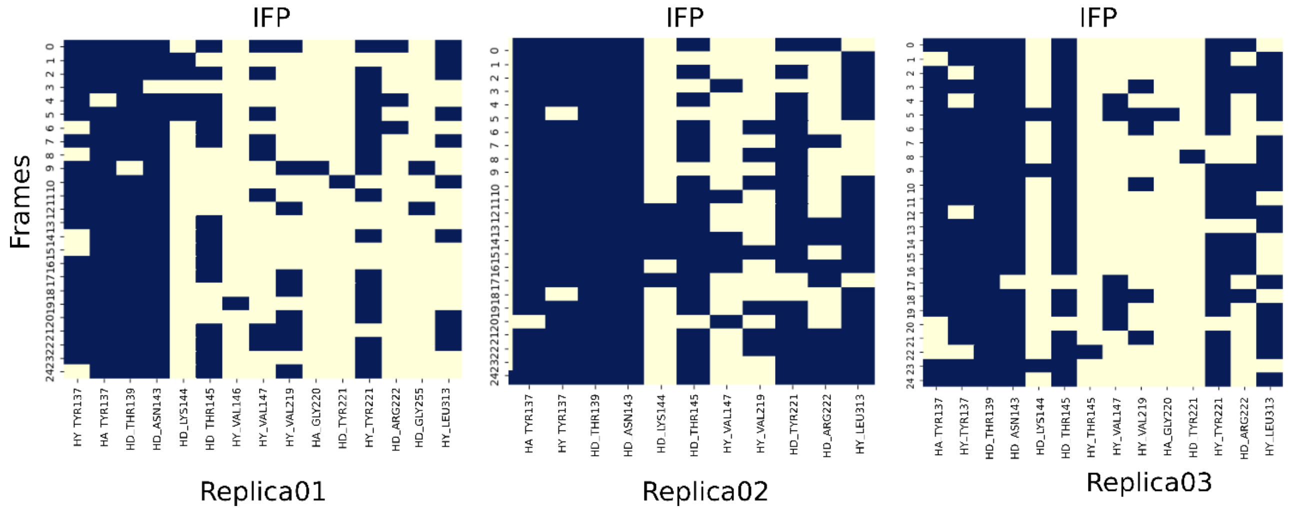

Supplementary Fig. S2 Stability of the modelled UniPR1331-VEGFR2-D2-D3 complex during MD simulations. The RMSD is shown as a function of time for the simulation of each replica for VEGFR2-D2-D3 (black lane) and UniPR1331 (red lane). RMSD of VEGFR2 was calculated on C-α atoms of D2 and D3 (including the hinge region). That of UniPR1331on C-α, oxygen and nitrogen atoms.

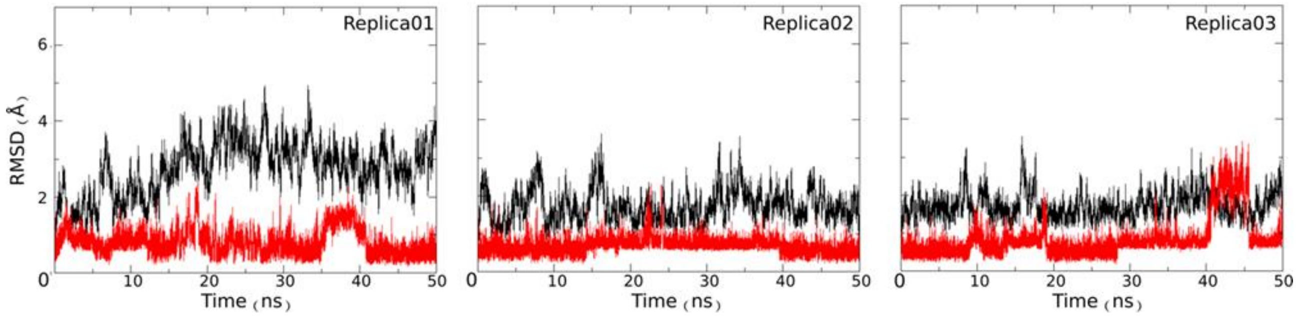

Supplementary Fig. S3 Superimposition of the D2-D3 domain of VEGFR2 in complex with UniPR1331 and VEGF. VEGFR2 and VEGF are depicted in cartoons representation with key residues shown in sticks representations (green and white, respectively). UniPR1331 is depicted in magenta sticks colored by atoms.

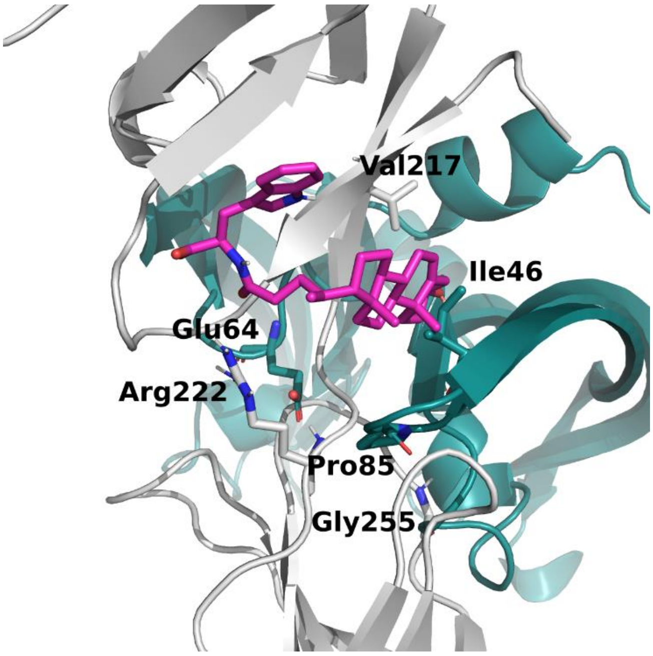

Supplementary Fig. S4 Original WB analysis used to generate Fig. 3 in which lanes have been cropped and reorganized to allow a direct comparison of the same experimental conditions in the different cell lines.

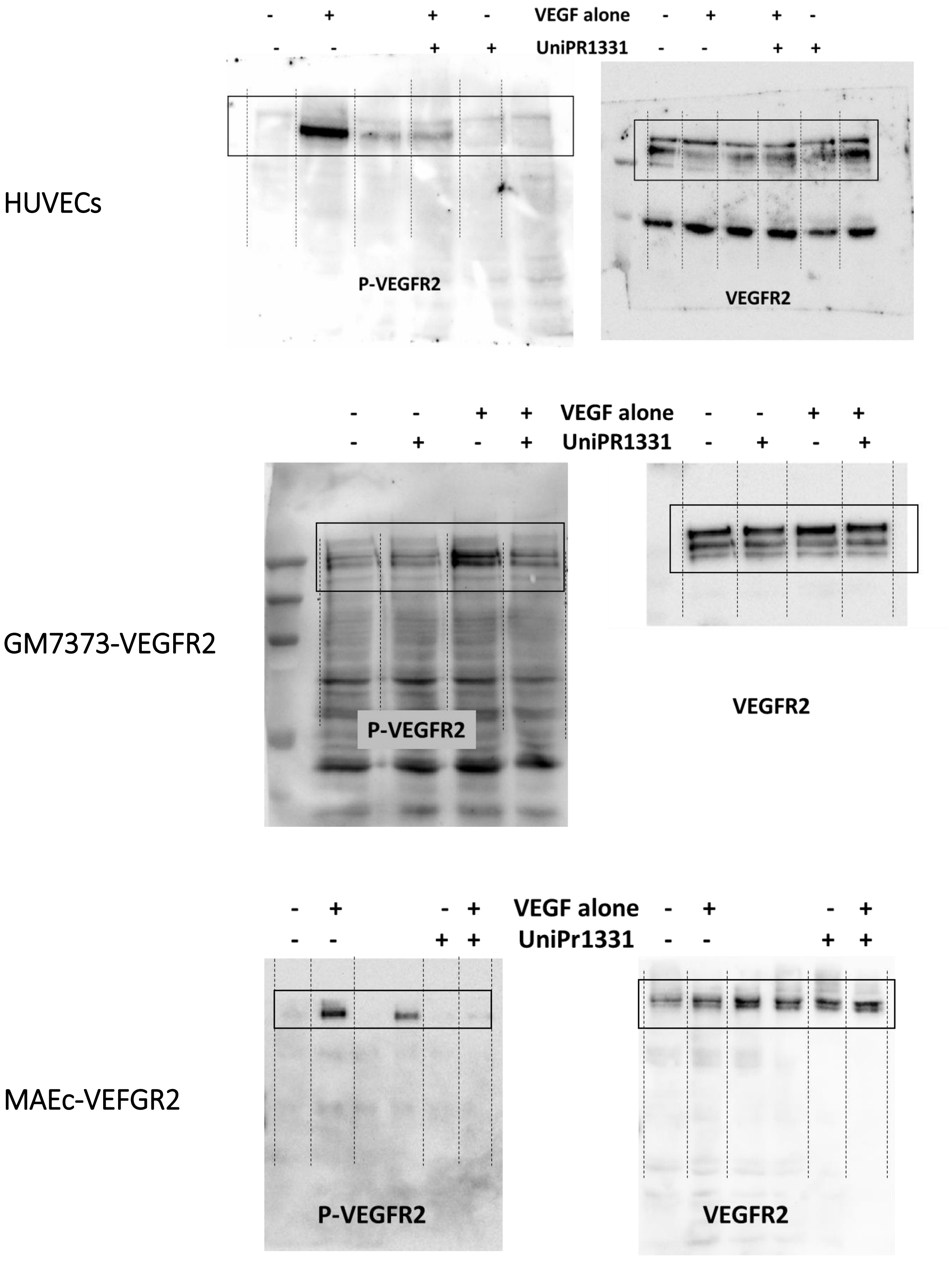

Supplementary Fig. S5 Original WB analysis used to generate comparison of P-$ERK_{1/2}$ in different cell lines in Fig. 4.

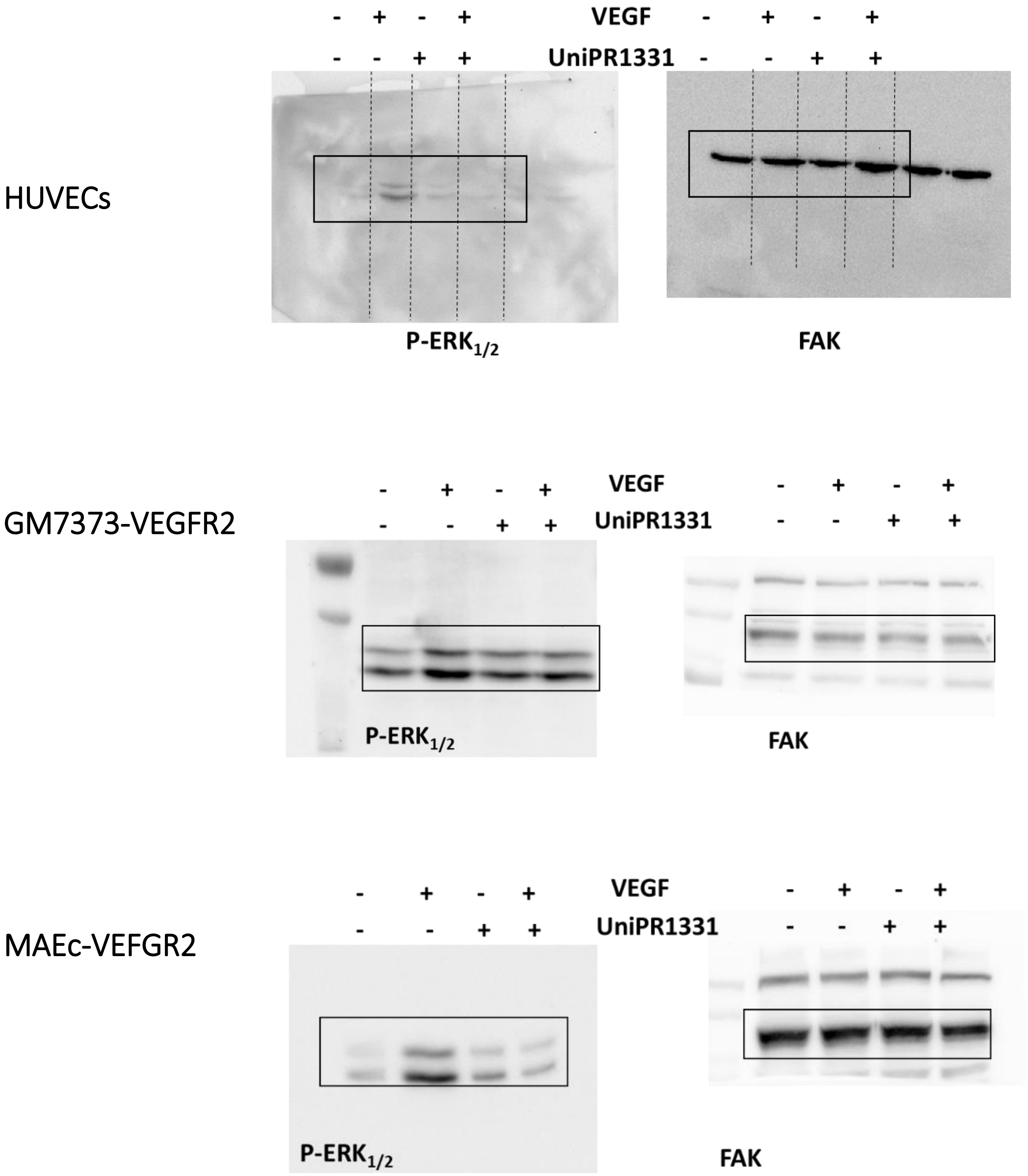

Supplementary Fig. S6 Absence of a cytotoxic effect of UniPR1331. HUVECs were incubated for 2 or 16 h with increasing concentration of UniPR1331 and evaluated for the release of LDH. Data are normalized to 0.5% DMSO considered as 0% LDH release and to 0.5% Triton considered as 100% LDH release.

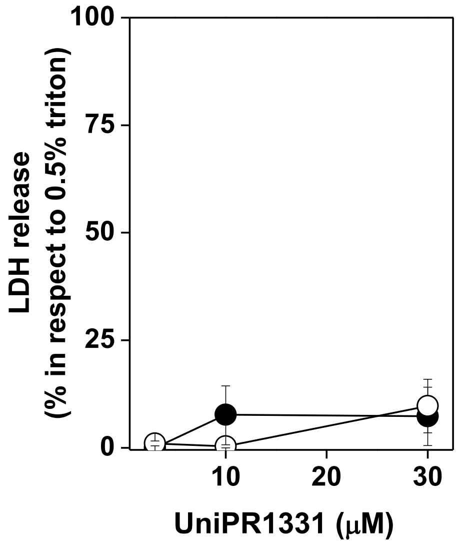

Supplementary Fig. S7 Evaluation of the angiogenic response in zebrafish of DMSO or UniPR1331 injected in the perivitelline space. Quantification of AP+ ectopic sprouts are expressed as total sprout length/embryo expressed in μm. The number of embryos analysed are embedded in the figure (ns: not significant).

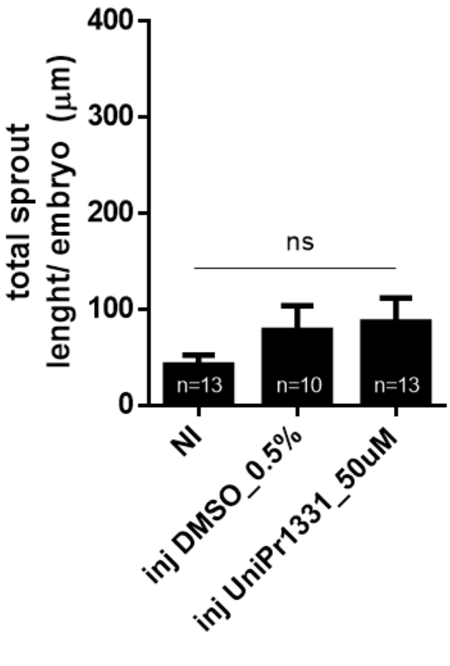

Supplementary Fig. S8 Effect of TNYL-RAW and Dynasore on VEGFR2 activation. HUVECs were stimulated with 10 ng/ml VEGF and TNYL-RAW or Dynasore (both at 50 µM) in the absence or in the presence of UniPR1331 (30 µM). Then, cells were analyzed by WB with anti-P-VEGFR2 antibody. Uniform loading was confirmed with anti-FAK antibody. The results shown are representative of another one that gave similar results.

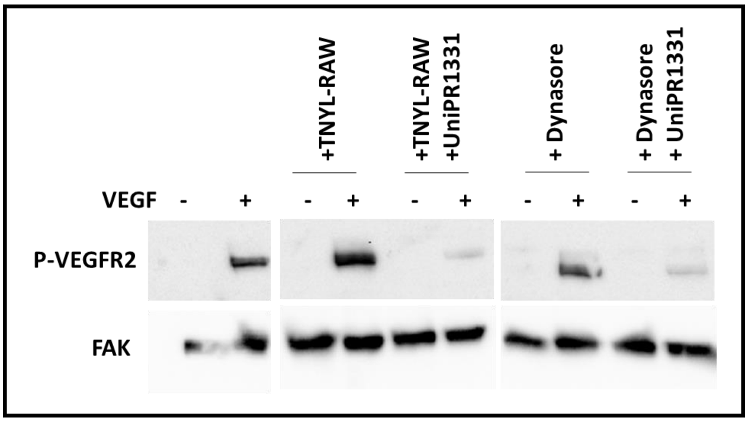

Supplement: Supplementary file 1 — Supplemental material [file 41417_2021_379_MOESM1_ESM.pdf]
